# Melatonin Supplementation and Anthropometric Indices: A Randomized Double-Blind Controlled Clinical Trial

**DOI:** 10.1155/2021/3502325

**Published:** 2021-08-10

**Authors:** Salman Mohammadi, Reza Rastmanesh, Farzaneh Jahangir, Zohreh Amiri, Kurosh Djafarian, Mohammad Ali Mohsenpour, Soheil Hassanipour, Ali Ghaffarian-Bahraman

**Affiliations:** ^1^Department of Clinical Nutrition and Dietetics, National Nutrition and Food Technology Research Institute, Shahid Beheshti University of Medical Sciences, Tehran, Iran; ^2^Independent Researcher, Iran; ^3^Department of Community Nutrition, School of Nutritional Sciences and Dietetics, Tehran University of Medical Sciences, Tehran, Iran; ^4^National Nutrition and Food Technology Research Institute, Faculty of Nutrition and Food Technology, Shahid Beheshti University of Medical Sciences, Tehran, Iran; ^5^Department of Clinical Nutrition, School of Nutritional Sciences and Dietetics, Tehran University of Medical Sciences, Tehran, Iran; ^6^Department of Clinical Nutrition, School of Nutrition and Food Sciences, Shiraz University of Medical Sciences, Shiraz, Iran; ^7^Student Research Committee, Shiraz University of Medical Sciences, Shiraz, Iran; ^8^Gastrointestinal and Liver Diseases Research Center, Guilan University of Medical Sciences, Rasht, Iran; ^9^Occupational Environment Research Center, Rafsanjan University of Medical Sciences, Rafsanjan, Iran

## Abstract

Obesity, as the most common metabolic disorder in the world, is characterized by excess body fat. This study is aimed at determining the effects of melatonin supplementation on body weight, nody mass index (BMI), waist circumference (WC), and body fat mass percentage (BFMP) in people with overweight or obesity. Thirty eight overweight or class-I obese adult individuals were recruited in the study (8 men and 30 women). Participants prescribed a weight-loss diet and then randomly were allocated to melatonin or placebo groups. Participants received either a 3-milligram melatonin or placebo tablet per day for 12 weeks. In order to assess differences at the significance level of 0.05, repeated measure ANOVA and paired *t*-test were used. According to the results, a significant reduction was found in participants' body weight, WC, and BMI in both groups (*p* = 0.001). However, for the last six weeks, significant reductions of these parameters were observed only in the melatonin group (*p* = 0.01). The BFMP of participants in the melatonin group showed a significant reduction at the end of the study compared to the initial measurements (*p* = 0.008). Nevertheless, the results of the present study alone are not sufficient to conclude on the effects of melatonin consumption on anthropometric indices, and it seems that further studies are required in this regard.

## 1. Introduction

Obesity is a major public health problem that has swept the world, especially during the last decades [1]. According to the epidemiological studies, it is predicted that 85% of the US adult population will be overweight, and 50% will exceed the obesity criteria by 2030 [2]. Several epidemiological studies have established a strong association between obesity and the development of some disorders including hypertension, diabetes mellitus, coronary heart disease, cancer, and mental disorders [3]. Although many hypotheses have evolved to explain the causation for obesity, no single theory can explain all manifestations or apply consistently to all individuals. There are at least two main causes for the obesity's high global prevalence: (1) decreasing people's energy expenditure due to automobilism, increasing screen time, and communization of low-calorie-consuming office occupations and (2) growing desire for consumption of calorie-dense food items mainly as a result of intensive advertisement [4].

To date, lots of efforts have been spent to curb the incidence of obesity. Dietary management, promotion of physical activity [5], and medication therapy [6] are among the most important interventions. However, the current strategies lack the desired outcomes, since the obesity is spreading rapidly worldwide [7]. It is shown that melatonin secretion level can affect the weight gain process [8]. Melatonin, which is chemically referred to as N-acetyl methoxy-tryptamine, is a naturally occurring compound found in animals, plants, and microbes [9]. In mammals, melatonin is secreted into the bloodstream by the pineal gland, particularly during dark times [10]. Given that the exposure to artificial blue light can suppress melatonin secretion [11], people living in modern societies are much more exposed to artificial light than their ancestors, partially as a result of which the prevalence of obesity is increasing nowadays [8].

Melatonin is also commercialized in many countries as a dietary supplement. These supplements are reported to have low or no adverse effects [12]. Although there is a growing trend in the number of melatonin-related human studies in recent decades [8, 13–16], a limited number of them have evaluated the effects of melatonin on anthropometric indices, some of which are contradictory in their findings. Thereby, in the present study, we aimed to determine the effects of melatonin supplementation on body weight and BMI, WC, and BFMP of patients with overweight or class-I obesity.

## 2. Materials and Methods

### 2.1. Design and Protocol

The present study was a blocked randomized double-blind controlled clinical trial, and its protocol was registered in the Iranian Registry of Clinical Trials (IRCT, http://www.irct.ir) with the following registration code: IRCT2015020120891N1.

### 2.2. Participants

Patients were recruited from overweight (25 ≤ BMI < 29.9) or class-I obese (30 ≤ BMI < 35) adult individuals, ageing between 19 and 55 years, not suffering from metabolic disorders including diabetes mellitus and thyroid disorders who referred to SBMU Clinic of Nutrition and Diet Therapy, Tehran, Iran. Participants were excluded if they were pregnant, lactating, or consuming weight affecting agents including glucocorticoids, anticonvulsions, antidepressants, and contraceptive drugs. All eligible participants signed an informed consent at the beginning of the study.

### 2.3. Randomization, Blinding, and Concealment

Participants were randomly assigned into two groups, namely, the melatonin or the placebo. Although melatonin and placebo tablets were apparently similar; they were placed into identical containers with different labels (A or B) by an independent researcher in order to blind both participants and interviewers during the intervention. Additionally, 4 random blocks of 11 individuals were created using random allocation software (version 1.0, Isfahan, Iran). These blocks were then randomly placed in two gender strata to provide an equal proportion of men and women.

For concealment, all the predicted A/B codes were printed in pieces of paper and were separately placed into envelopes in the sequence of randomized blocks. Then, two boxes labeled for men and women were used as containers for sequenced envelopes. The lids of the boxes were designed in such a way that only the top envelope could be removed at a time. The first envelope taken out of the women's box determined the interventional group for the first female participant. The same process applied to male clients. This procedure curbed the bias of interviewers, since they were unaware about the patients' group till the interview ended. Also, the interviewers could not predict the next clients' group of intervention. Based on group allocation, participants consumed either a 3-milligram melatonin or a placebo tablet (ESI, Italy), two hours before going to bed for 12 weeks.

### 2.4. Measurements

In the present study, participants completed a general information questionnaire upon arrival. Being eligible based on aforementioned inclusion criteria, patients were evaluated for anthropometrics and interviewed to complete 24-hour food recalls and physical activity questionnaire (IPAQ). Then, patients were asked not to change their lifestyle in terms of physical activity, dietary intakes, and working conditions in two weeks before the intervention (run-in period).

### 2.5. Anthropometrics

Participants' body weight and heights were measured with the least possible clothing and barefooted using a stadiometer with the accuracy of 100 grs and 0.5 cm (Seca, Hamburg, Germany). Height was recorded while the individuals' heads were in Frankfurt position [17]. In addition, WC was measured at the midpoint of the abdomen located between the 12^th^ rib and the iliac crest using a nonstretching measuring tape [18]. BMI then was calculated using standard Quetelet's equation, i.e., (weight in kgs)^/^(height in m)^2^ [19]. Also, body composition was assessed by Bodystat (Quad Scan 4000, England).

### 2.6. Physical Activity

International physical activity questionnaire with 15 questions (9 non-occupational and 6 occupational related activities) was used to assess physical activity score of participants. This questionnaire was validated previously [20], and its validity and reliability were approved in Iran [21].

### 2.7. Dietary Intake Analyses

Three 24-hour food recalls were collected during one week before the intervention, and all participants were prescribed a low calorie diet; then, these interviews were repeated at the end of the sixth and twelfth weeks to finally collect a total of nine recalls from each participant. Energy requirement for patients was calculated by Harris Benedict equation using the weight contributing to the BMI of 22 kg.m^−2^ [22]. In order to achieve a low calorie diet, 500 kcal was reduced from calculated energy requirement. Patients were asked to maintain their usual lifestyle and physical activity during the study [23]. Dietary intakes were analyzed using Nutritionist-IV software version 4.0 (1995, First databank, San Bruno, California, USA), modified to Iranian food items, in order to assess the intake of total calorie, macronutrients, micronutrients, and fiber [24].

### 2.8. Body Melatonin Level

In order to evaluate the body melatonin level, participants were asked to collect two saliva specimens, at midnight and in the morning of the first and last day of intervention, and then immediately place them in the freezer to be delivered to the researchers as soon as possible. The collected samples were stored in -81°C freezer until the study ended. Saliva melatonin level was measured using the ELISA kit assay (IBL, Germany).

### 2.9. Data Analysis

Data were analyzed with SPSS statistical software, version 17. The normality of quantitative data was controlled using the Kolmogorov-Smirnov test. Qualitative variables were compared between two groups using the *χ*^2^ test and repeated measure ANOVA or its nonparametric equivalent; Freidman's test was used to compare the means of quantitative variables. Furthermore, within group differences were assessed using the paired *t*-test (or Wilcoxon signed ranks test), whilst Student's *t*-test (or Mann–Whitney-*U* test) was used for between groups analyses. In the present study, *p* value less than 0.05 was considered as significant.

## 3. Results

**Figure 1:**
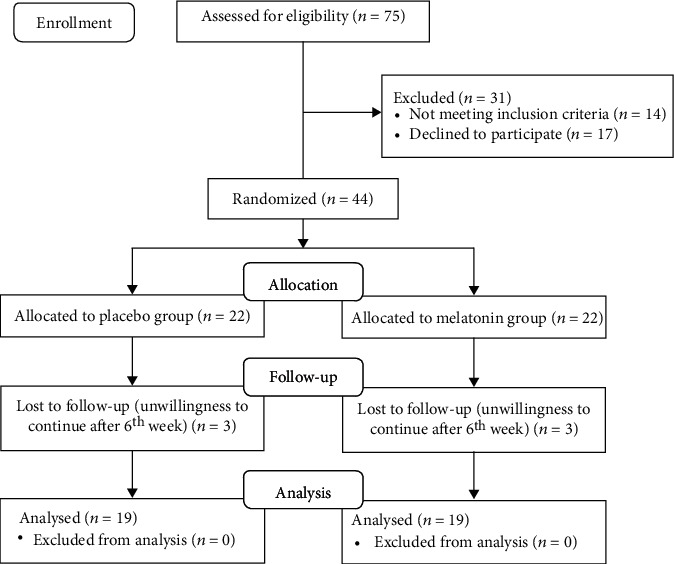
CONSORT flow diagram demonstrates the study procedure.

Among 75 screened individuals, 14 were excluded due to lack of eligibility. Furthermore, 17 eligible patients were not willing to continue participation. Therefore, 44 subjects included in the study (*n* = 22 in melatonin and *n* = 22 in placebo group). After six weeks, a total of six participants left the study, and both groups were the same in losses (Figure 1). Thus, 19 participants in each group (86%) finished the study. Participants' mean age was 38.95 ± 11.63 and 37.84 ± 11.38 for the melatonin and placebo group, respectively (*p* value =0.77). Despite the gender-based stratification, groups were not exactly the same in the proportion of female/male, since two out of three lost to follow-ups in the melatonin group were female, whilst all were male in the placebo group. However, between-group difference was not statistically significant in this regard (*p* value = 0.36). In addition, no significant differences were seen between groups for education, marital status, and smoking (Table 1). According to the findings, menopausal status for female participants did not show significant difference between groups (five vs. four postmenopausal women in the melatonin and the placebo groups, respectively, *p* = 0.596).

As shown in Table 2, total energy intake showed a significant reduction during the study in both groups (*p* = 0.001). However, this difference was not significant between the 6^th^ and the 12^th^ weeks. The overall results were the same for other dietary factors (*p* > 0.05). According to our findings, weight and BMI reduced significantly at 12^th^ week in the both groups. In addition, mean WC of participants at 6^th^ week was significantly lower than its initial amount in the both groups. Meanwhile, comparing the values between the 12^th^ and the 6^th^ weeks, patients' mean WC was measured lower and unchanged in the melatonin and the placebo groups, respectively (Table 3). Furthermore, BFMP at the end of study was observed to be lower than its baseline amount only in the melatonin group. All of the anthropometric changes were occurred while the participants of both groups did not significantly change their physical activity levels throughout the interventional periods.

As observed in Table 3, the total daily sleep hours remained unchanged in both groups during the study. Moreover, our findings indicated that the saliva melatonin levels at the end of the study were not statistically different from the baseline amounts in the both groups. Furthermore, we also found no difference between the nocturnal and the diurnal saliva melatonin levels comparing the final and the baseline measurements (Table 3).

## 4. Discussion

In the present study, we observed a significant reduction in body weight and BMI in the both melatonin and placebo groups. Furthermore, WC reduced in the both groups during the study. However, this reduction was only significant in the melatonin group while comparing the middle and the final values. In addition, we found that BFMP lowered significantly only in melatonin group.

The association between melatonin, obesity, and metabolic disorders has been widely investigated in animal models [25–29]. To the best of our knowledge, few human investigations have been conducted in this regard. In a study carried out by Kozirog et al. [30], administration of melatonin (5 mg/day) for one month reduced body weight and BMI in patients with metabolic syndrome. However, two highly determining factors including dietary energy intake and physical activity level were not controlled in Kozirog's study.

In addition, data from the Vitamins and Lifestyle Cohort Study of western Washington [31] with a total of 15655 participants indicated that melatonin supplementation was related to weight loss and less weight regain in adults. Also, findings of a clinical trial showed that treatment with a standard balanced diet (1500 kcal) and 5 mg/day of melatonin for 24 weeks could significantly reduce body weight and BMI in postmenopausal women, whilst it was ineffective on waist circumference [32]. However, this study had some considerable limitations including prescription of a same diet for all participants regardless to their body weights, energy requirements, and physical activity levels.

In general, the main mechanism by which melatonin affects energy metabolism and reduces body weight is presumably complex and obscure [33]. Nevertheless, melatonin is shown to increase the capacity of nonshivering thermogenesis via brown adipose tissue (BAT) activation in small mammals [34]. BAT is a unique type of adipose tissue that is exclusively found in mammalian species [35]. The function of BAT is primarily to produce heat (nonshivering thermogenesis) for adaptation to marked ambient temperature changes, especially in cold environments. Regardless to the pathways, mitochondrial uncoupling protein-1 (UCP1) plays a major role in BAT-mediated thermogenesis. UCP1 (also known as Thermogenin) presents in brown adipocytes in substantial amounts. This protein uncouples the oxidation process from ATP formation in the mitochondrial electron transport pathway by letting protons leak through the inner membrane of mitochondria and reducing the main force for ATP synthesis, i.e., *trans*-membrane electrochemical proton gradient [36]. Consequently, the dissipated proton motive force is used for heat production, namely, nonshivering thermogenesis [8]. Investigating the effects of NEU-P11 (also called Ramelteon) as a potent selective MT1/MT2 receptor agonist, it is suggested that melatonin receptor-mediated pathways may play an important role in the relationship between melatonin and obesity [37, 38], particularly that MT receptors present in BAT in abundance [8]. However, the important role of sympathetic nervous system's activation via hypothalamic receptors and its subsequent lipolysis in thermogenesis is not negligible [25].

According to our results, a significant reduction in body weight and BMI was observed in both groups after the intervention period. This may be due to the participants' weight loss diet follow-up in the both groups. However, there was no between-group significant difference in body weight comparing the middle and the final values. This is while the participants properly continued following their tailored weight loss diets during the second half of the study, and no change was observed in energy intake and physical activity level in this period. This finding supports the hypothesis of calorie scarcity adaptation, according to which body stands against further weight loss, mainly via diminishing its basal metabolic rate [39].

Despite the aforementioned animal studies, our findings indicated that melatonin supplementation had no effects on body weight and BMI, since weight loss was experienced by either melatonin or placebo participants. However, biological and physiological diversities existing in different species have to be taken into consideration. For instance, BAT mass suggesting to be responsible for melatonin weight-affecting properties differs substantially from species to species [8].

Nevertheless, a recent meta-analysis on clinical trial studies showed that daily melatonin intake did not impact anthropometric parameters including body weight, BMI, and WC [40]. One of the studies included in this meta-analysis is a study conducted in 2015 in Poland. In this study, it was found that daily consumption of 5 mg of melatonin along with 20 mgs of fluoxetine significantly reduced BMI in postmenopausal women [41]. It seems that this study should have been excluded from the meta-analysis due to the simultaneous administration of melatonin and fluoxetine. In addition, another study found that 10 mg per day melatonin supplementation for one month failed to cause significant changes in anthropometric indices including weight, BMI, and WC [42]. However, the results of this study were not included in the abovementioned meta-analysis.

Moreover, among the studies included in this meta-analysis, only one study indicated waist circumference reducing effects of melatonin [43]. In this study, melatonin was prescribed to menopausal women along with myoinositol; thereby, it appears that it was not eligible to be included in the meta-analysis. Nonetheless, we found that daily consumption of 3 milligrams of melatonin could significantly reduce WC in overweight and obese people. It seems that the findings of this meta-analysis probably change if the results of the present study and other missed investigations are included, and the noneligible studies are excluded.

Moreover, the results of our study indicated that BFMP of participants in the melatonin group was significantly lower than their initial amounts, while this difference was not significant in the placebo group. This finding suggests that besides the administered dosage of melatonin, consumption duration is a determining factor in its effectiveness, since consumption of 3 milligrams of melatonin per day for 6 weeks could not change BFMP, while a statistically significant decrease was observed after 12 weeks of supplementation. This finding was supported by a crossover animal study carried out by Hasson et al. [44]. According to this study, abdominal fat mass of melatonin receiving rats (0.4 microgram melatonin per milliliter of consuming water) was lower than the comparison group. However, the plasma level of melatonin did not show difference between the two groups in this study.

In addition to experimental studies, similar conclusions are achieved by human investigations. A Japanese cross-sectional investigation indicated that exposure to more than 3 luxes of artificial light at night had a positive correlation to higher waists circumference [45]. Furthermore, in a clinical trial study, it was found that body fat mass of postmenopausal women with osteopenia after one year of consumption of 1 to 3 mg of melatonin per night was lower than the comparison group [46]. Another study performing on patients with bipolar disorder and schizophrenia had similar results and found that short-term (two months) consumption of melatonin at a dose of 5 mg per day could significantly reduce body fat mass [47]. However, in neither of these studies, the two main confounding variables including energy intake and level of physical activity were taken into consideration.

Simultaneous reduction in WC and BFMP along with the stabilization of body weight in the melatonin group suggests that waist fat mass may partially replace by lean mass including bone mineral density and muscle mass. This finding is also supported by previous investigations. It is believed that melatonin directs mesenchymal stem cells towards osteogenesis while inhibiting dipogenesis in human [46, 48]. Regardless to the mechanisms, however, WC reduction in people with overweight or obesity is in much importance, since it has been established that abdominal obesity predicts obesity-related health risks more accurate than some of other anthropometric indices including BMI [49–51].

In the present study, we observed that the salivary levels of melatonin at the end of the study were higher and lower than the baseline values in the two groups of melatonin and placebo, respectively; although, these differences were not statistically significant. However, it is worth noting that there are several components affecting statistical significance, including sample size. Thereby, it is suggested that statistical significance should always be considered along with clinical significance [52]. The findings presented in Table 3 showed that the melatonin salivary levels in the two groups had changed by more than 25% of initial values in some comparisons, but were still not statistically significant. Although the variables related to nocturnal and diurnal levels of saliva melatonin did not have the necessary assumptions for conduction of parametric tests, it seems that the statistical significance of observed differences would be changed, if we had a larger sample size in this study.

Despite the appropriate design in which issues such as stratified-blocked randomization, double blindness, and concealment were considered, we encountered some limitations in the present study. In this study, men participated less than women, and this made the gender ratio in favor of women in the both groups. However, to eliminate this, we conducted a gender-stratified blocked random selection from the very beginning of the study. In addition, melatonin was prescribed in an identical dose to the participants, regardless to their initial body weight, since the only available form of this supplement were 3-milligram tablets. Thereby, it is recommended to the researchers to use weight-dependent doses of melatonin in their investigations. We also found that making significant changes in anthropometric parameters require different durations. Therefore, it seems that further studies with strong designs are needed to optimize the duration of the intervention.

Moreover, basal metabolic rate, daily energy intake, and physical activity level, which are very influential on anthropometric indices, were measured by the conventional methods of a questionnaire in the present study. Thereby, it is highly recommended to use more accurate methods such as calorimeters and electrical activity meters for future studies. In addition, it is shown that melatonin affects body weight and body fat mass by altering BAT activity, and we suggest that the activity level of this tissue be monitored during the intervention if modern instruments are available, including positron emission tomography scan technology.

## 5. Conclusions

We observed that the melatonin supplementation at the dosage of 3 milligrams per day for 3 months did not make significant changes in weight and BMI, but significantly reduced WC and BFMP.

## Figures and Tables

**Table 1:** Baseline characteristics of the participants.

		Group		*p* value^#^
		Melatonin (*n* = 19)	Placebo (*n* = 19)	
Gender^∗^	Female	16 (84%)	14 (74%)	0.364
	Male	3 (16%)	5 (26%)	
Smoking	Smoker	1 (5%)	1 (5%)	0.757
	Nonsmoker	18 (95%)	18 (95%)	
Education level	Diploma and bellow	8 (42%)	12 (63%)	0.194
	Associate and higher	11 (58%)	7 (37%)	
Marital status	Married	14 (74%)	13 (68%)	0.721
	Single	5 (26%)	6 (32%)	

^∗^Data are shown as *n* (%). ^#^ Chi-square statistical analysis was used. *p* value <0.05 considered as significant.

**Table 2:** Mean ± standard deviation of participants' dietary intakes during the study.

Outcome	Group	Time			*p* value
		Baseline	6^th^ week	12^th^ week	
Calorie (kcal/day)	Melatonin	2055.72 ± 261.61^a^	1586.83 ± 281.21^b^	1624.58 ± 324.25^b^	0.001^†^
	Placebo	2112.23 ± 243.43^a^	1558.47 ± 19825^b^	1558.21 ± 213.33^b^	0.001^‡^
	*p* value	0.49^#^	0.83^##^	0.92^##^	
Carbohydrate (g/day)	Melatonin	2267.76 ± 34.77^a^	209.38 ± 39.24^b^	215.00 ± 38.67^b^	0.001^†^
	Placebo	273.86 ± 31.18^a^	201.75 ± 24.56^b^	200.55 ± 27.80^b^	0.001^‡^
	*p* value	0.57^#^	0.94^##^	0.36^##^	
Protein (g/day)	Melatonin	92.09 ± 11.13^a^	72.69 ± 14.00^b^	70.74 ± 14.58^b^	0.001^†^
	Placebo	94.47 ± 13.16^a^	69.48 ± 10.95^b^	71.21 ± 11.13^b^	0.001^‡^
	*p* value	0.55^#^	0.44^#^	0.25^##^	
Fat (g/day)	Melatonin	68.94 ± 8.88^a^	53.57 ± 10.87^b^	53.62 ± 9.64^b^	0.001^†^
	Placebo	70.86 ± 7.82^a^	52.24 ± 6.60^b^	52.42 ± 7.10^b^	0.001^‡^
	*p* value	0.48^#^	0.67^##^	0.89^##^	
Fiber (g/day)	Melatonin	15.44 ± 1.82^a^	12.18 ± 1.99^b^	12.60 ± 2.02^b^	0.001^†^
	Placebo	16.36 ± 3.01^a^	12.25 ± 1.46^b^	14.21 ± 7.87^b^	0.001^†^
	*p* value	0.26^#^	0.89^#^	0.40^##^	
Cholesterol (mg/day)	Melatonin	205.05 ± 69.66^a^	150.37 ± 89.04^b^	135.76 ± 89.64^b^	0.001^†^
	Placebo	187.73 ± 59.14^a^	136.21 ± 77.06^b^	110.56 ± 61.44^b^	0.001^†^
	*p* value	0.41^#^	0.31^##^	0.52^##^	
SFA (g/day)^∗^	Melatonin	20.59 ± 3.91^a^	15.79 ± 3.01^b^	16.80 ± 4.15^b^	0.001^†^
	Placebo	19.21 ± 2.33^a^	13.98 ± 2.37^b^	14.16 ± 1.96^b^	0.001^‡^
	*p* value	0.34^##^	0.085^##^	0.071^##^	
MUFA (g/day)	Melatonin	24.32 ± 3.58^a^	20.04 ± 6.34^b^	18.67 ± 4.29^b^	0.001^‡^
	Placebo	27.27 ± 4.55^a^	17.92 ± 3.88^b^	18.17 ± 4.62^b^	0.001^‡^
	*p* value	0.03^#^	0.22^#^	0.73^#^	
PUFA (g/day)	Melatonin	24.03 ± 3.69^a^	17.73 ± 3.01^b^	18.15 ± 2.87^b^	0.001^‡^
	Placebo	24.38 ± 4.13^a^	20.33 ± 3.18^b^	20.12 ± 3.07^b^	0.001^‡^
	*p* value	0.78^#^	0.18^#^	0.068^#^	

^∗^SFA: saturated fatty acid; MUFA: monounsaturated fatty acid; PUFA: polyunsaturated fatty acid. ^a,b^Similar letter in two-sided comparisons of a row indicates no significant difference. ^†^Nonparametric Friedman test. ^‡^Repeated measure ANOVA. ^#^Independent sample *t*-test. ^##^Nonparametric Mann–Whitney *U* test. ^∗∗^*p* value less than 0.05 is considered as significant.

**Table 3:** Mean and standard deviation of the study outcomes.

Outcome	Group	Time			*p* value
		Baseline	6^th^ week	12^th^ week	
Weight (kg)	Melatonin	82.54 ± 11.63^a^	80.69 ± 12.16^b^	80.32 ± 12.48^b^	0.001^†^
	Placebo	78.75 ± 9.83^a^	75.73 ± 9.35^b^	75.16 ± 9.53^b^	0.001^‡^
	*p* value	0.35^##^	0.28^##^	0.24^##^	
BMI (kg/m^2^)	Melatonin	31.01 ± 2.04^a^	30.29 ± 1.96^b^	30.14 ± 2.03^b^	0.001^‡^
	Placebo	30.40 ± 1.62^a^	29.24 ± 1.70^b^	29.02 ± 2.05^b^	0.002^‡^
	*p* value	0.30^#^	0.08^#^	0.10^#^	
Waist circumference (cm)	Melatonin	96.47 ± 9.36^a^	92.63 ± 8.90^b^	90.79 ± 9.20^c^	0.001^†^
	Placebo	93.74 ± 9.80^a^	90.16 ± 8.27^b^	89.37 ± 8.17^b^	0.001^‡^
	*p* value	0.65^##^	0.38^#^	0.62^#^	
Body fat mass (%)	Melatonin	40.43 ± 5.83^a^	38.95 ± 6.54^a,b^	39.10 ± 5.21^b^	0.196^†^
	Placebo	38.21 ± 7.72^a^	37.34 ± 7.56^a^	37.09 ± 8.53^a^	0.234^†^
	*p* value	0.64^##^	0.55^##^	0.39^#^	
Physical activity score (met.h/day)	Melatonin	38.51 ± 6.41^a^	39.75 ± 5.43^a^	40.8 ± 5.59^a^	0.065^†^
	Placebo	39.29 ± 6.16^a^	42.77 ± 6.54^b^	40.64 ± 4.92^b,a^	0.128^†^
	*p* value	0.54^##^	0.14^##^	0.93^#^	
Total daily sleep (hour)	Melatonin	7.38 ± 1.29^a^	7.68 ± 1.01^a^	7.63 ± 1.33^a^	0.229^‡^
	Placebo	7.31 ± 1.13^a^	7.31 ± 0.92^a^	7.31 ± 0.96^a^	0.172^†^
	*p* value	0.87^#^	0.21^##^	0.41^#^	
Nocturnal saliva melatonin (pgr/mL)	Melatonin	18.7 ± 17.44	—	21.7 ± 18.07	0.629^∗^
	Placebo	23.43 ± 22.35	—	16.98 ± 12.58	0.235^∗^
	*p* value	0.579^##^	—	0.620^##^	
Diurnal saliva melatonin (pgr/mL)	Melatonin	13.51 ± 13.71	—	17.4 ± 16.95	0.286^∗^
	Placebo	15.13 ± 13.43	—	16.36 ± 15.74	0.841^∗^
	*p* value	0.704^##^	—	0.737^##^	
Average saliva melatonin (pgr/mL)	Melatonin	16.49 ± 13.37	—	19.55 ± 14.79	0.44^∗^
	Placebo	20.03 ± 16.15	—	16.67 ± 12.19	0.63^∗^
	*p* value	0.63^##^	—	0.64^##^	

^a,b,c^The same letters in two-sided comparisons of a row indicate no significant difference. ^†^Nonparametric Friedman test. ^‡^Repeated measure ANOVA. ^#^Independent sample *t*-test. ^##^Nonparametric Mann–Whitney *U* test. ^∗^Wilcoxon signed ranks test. ^∗∗^*p* value less than 0.05 was considered as significant.

## Data Availability

Resources of data and statistical analysis outputs of this study can be provided by the author of correspondence.
